# Network Centrality of Resting-State fMRI in Primary Angle-Closure Glaucoma Before and After Surgery

**DOI:** 10.1371/journal.pone.0141389

**Published:** 2015-10-27

**Authors:** Fengqin Cai, Lei Gao, Honghan Gong, Fei Jiang, Chonggang Pei, Xu Zhang, Xianjun Zeng, Ruiwang Huang

**Affiliations:** 1 Department of Radiology, the First Affiliated Hospital of Nanchang University, Nanchang 330006, Jiangxi Province, China; 2 Center for the Study of Applied Psychology, Key Laboratory of Mental Health and Cognitive Science of Guangdong Province, School of Psychology, South China Normal University, Guangzhou 510631, Guangdong Province, China; 3 Department of Ophthalmology, the First Affiliated Hospital of Nanchang University, Nanchang 330006, Jiangxi Province, China; 4 Affiliated Eye Hospital of Nanchang University, Jiangxi Research Institute of Ophthalmology & Visual Sciences, Nanchang 330006, Jiangxi Province, China; Wake Forest School of Medicine, UNITED STATES

## Abstract

**Purpose:**

Using voxel-wise degree centrality (DC), as measured by resting-state fMRI, we aimed to study alterations in the brain functional networks in patients with primary angle-closure glaucoma (PACG) and to reveal the plastic trajectories of surgery.

**Methods:**

A total of 23 preoperative PACG patients (49.48 ± 14.37 years old) were recruited to undergo a resting-state fMRI scan, and 9 of them were rescanned 3 months after surgery. All PACG patients underwent a complete ophthalmologic examination, including intraocular pressure (IOP), retinal nerve fiber layer (RNFL) thickness, vertical cup to disc ratio (V C/D), and average cup to disc ratio (A C/D). Another 23 gender- and age-matched healthy controls (48.18 ± 9.40 years old) underwent scanning once for comparison. The group difference in DC was calculated in each voxel, and the correlations between the DC value and each of the clinical variables were analyzed in the PACG patients.

**Results:**

Preoperative PACG (pre-PACG) patients showed significantly decreased DC in the bilateral visual cortices but increased DC in the left anterior cingulate cortex (ACC) and caudate (*p* < 0.05, corrected) compared with the controls. Statistical analysis showed a significantly negative correlation between DC in the bilateral visual cortices and the IOP score and between DC in the anterior cingulate cortex (ACC) and both the A C/D and V C/D scores in the pre-PACG patients. Three months after surgery, these postoperative PACG (post-PACG) patients showed a significantly increased DC in both the bilateral visual cortices and the left precentral gyrus compared with the pre-PACG patients.

**Conclusions:**

Our results suggest that PACG may contribute to decreased functional centrality in the visual system and to increased degree centrality in cognition-emotional processing regions. Alterations in visual areas seem to parallel the cup to disc ratio, but not the duration of angle closure. The changes of functional centrality in PACG patients after operation may reveal the plasticity or degeneration of the visual-associated brain areas. Our findings may provide further understanding of the pathophysiology of PACG.

## Introduction

Glaucoma affects more than 70 million people worldwide and is the leading cause of irresistible blindness [1]. Based on the anatomy of the anterior chamber angle, glaucoma can be roughly divided into two main categories: primary open-angle glaucoma (POAG) and primary angle-closure glaucoma (PACG). POAG is characterized by the progressive death of retinal ganglion cells (RGCs) in association with elevated intraocular pressure (IOP). The death of RGCs can lead to the loss of neural cells in the entire visual pathway from the lateral geniculate nucleus (LGN) to the visual cortex. Previous evidence has indicated that Wallerian and transsynaptic degeneration play a causative role in POAG [2]. However, in China and other Asian countries, PACG accounts for more than one quarter of all primary glaucoma cases and for 80% of world PACG cases [3,4]. It is generally believed that PACG results from an elevated IOP as a consequence of iris-trabecular meshwork contact in the angle of the eye, a combination of predisposing anterior segment anatomy and unfavorable physiological behavior [5,6,7,8]. PACG is responsible for a substantial proportion of blindness cases in many Asian countries, and it has been estimated that PACG blinds proportionately more people than POAG does globally [9]. However, the neurological basis is largely unknown; the brain’s functional involvement and its contribution to blindness progression have not been fully characterized. Brain imaging may facilitate non-invasive exploration.

Previous neuroimaging studies demonstrate that glaucoma patients exist not only the morphometric changes along the visual pathway but also the functional alterations within the visual system and the visual associated areas. Structurally, the volume of the optic nerve (ON), optic chiasm, lateral geniculate nucleus (LGN) and associated brain areas [10,11], the thickness of visual cortices [12] were decreased detected with the high-resolution T1WI images in glaucoma patients. The diffusion tensor imaging (DTI) with the main parameters of fractional anisotropy (FA) and mean diffusivity (MD) also reveals the degeneration of the visual pathway [13,14,15], such as the FA decreases and MD increases in ON, optic tract (OT), optic radiation (OR) and other white matter areas. Functionally, the cerebral blood flow and the metabolite concentration of the visual cortices in glaucoma patients also exist alterations using arterial spin labeling fMRI [16] and magnetic resonance spectroscopy (MRS) [17], respectively.

Resting-state functional magnetic resonance imaging (rsfMRI) has increasingly emerged as a useful tool for studying the human brain functional connectome [18], which is thought to chart the circuits that underlie the physiological basis for information processing and mental representations [19]. Changes in POAG have been reported, including disrupted large-scale brain network coupling [14], decreased visual network functional connectivity [20], altered amplitude of low frequency fluctuations [21], and spontaneous brain activity changes with regional homogeneity analysis [22]. However, the underlying changes of PACG measured by rsfMRI have received little attention. Characterizing the functional connectomic alterations in PACG and the plastic trajectories of surgery would be critical steps in understanding the pathophysiological processes of PACG.

Voxel-wise degree centrality (DC) measures the topology of the architecture of the brain functional connectome at the voxel level [23]. DC represents the number of direct connections for a given voxel in the voxel-wise connectome. It requires no priori definition of regions of interest (ROIs) and can provide information about the functional connectivity within the whole-brain network. Previous studies have reported that it has relatively high test-retest reliability [24], and DC has been employed in a number of neurodegenerative and psychiatric disorders [24,25,26,27,28].

In this study, we sought evidence for potential functional connectomic plasticity of PACG patients before and after surgery. We hypothesized that (1) the visual-related DC of preoperative PACG patients would show a significant difference from that of the controls and (2) postoperatively, with the improvement of clinical symptoms, partial plastic changes may occur in the visual pathway.

## Methods

### 2.1. Subjects

Twenty-three preoperative PACG patients (8 M/15 F, age = 49.48 ± 14.37 years old) were recruited from February to October 2014 from the Department of Ophthalmology of the First Affiliated Hospital of Nanchang University, China. The inclusive criteria for the PACG patients were as follows: (1) narrow anterior chamber angle in one or two eyes determined by gonioscopy, (2) the characteristic optic disc damage (optic disc cupping or thinning), and (3) the corresponding glaucomatous visual field defects (tubular vision or central island). All of the patients underwent a detailed ophthalmology examination, including the state of the anterior chamber angle determined by the gonioscopy, intraocular pressure (IOP) measured using a tonometer, the optic disc changes evaluated with the optical coherence tomography (Cirrus HD-OCT) [29], and the vision function measured with the Peripheral Vision Test (Humphrey Field Analyzer, Humphrey HFA II-i). Exclusion criteria for the patients were those who (1) had secondary glaucoma or any other ocular disorders that could affect the optic visual pathway; (2) had neural-associated diseases or chronic pains, hypertension, diabetes, or a history of brain surgery; or (3) were unable to attend MRI scanning due to metal implantation or a history of claustrophobia or other psychological disorders. In addition, we recruited 23 age- and gender-matched healthy subjects as controls. All of the subjects were right-handed, according to their self-reports. The study was conducted in accordance with the Declaration of Helsinki. The study protocol was approved by the Institutional Review Board of the First Affiliated Hospital of Nanchang University. Written informed consent was obtained from each subject prior to the study. Table 1 lists the demographic information of the pre-PACG patients and controls.

**Table 1:** Demographics and clinical characteristics of PACG patients and controls.

	PACG	Controls	*p-*value
Age (years old)	49.48 ± 14.37	48.18 ± 9.40	0.721
Sex (M/F)	8/15	8/15	> 0.99
Handedness	23R	23R	> 0.99
Duration of disease, day	2 d-2920 d, mid 180 d	-	-
RNFL(μm)	79.74 ± 20.87	-	-
Average C/D	0.68 ± 0.19	-	-
Vertical C/D	0.65 ± 0.20	-	-
IOP (mmHg)	39.00 ± 11.09	-	-

Abbreviation: RNFL, retina nerve fiber layer; A C/D, average cup to disc ratio; V C/D, vertical cup to disc ratio; IOP, Intraocular pressure; male (M), female (F), day (d), right (R).

**Fig 1:**
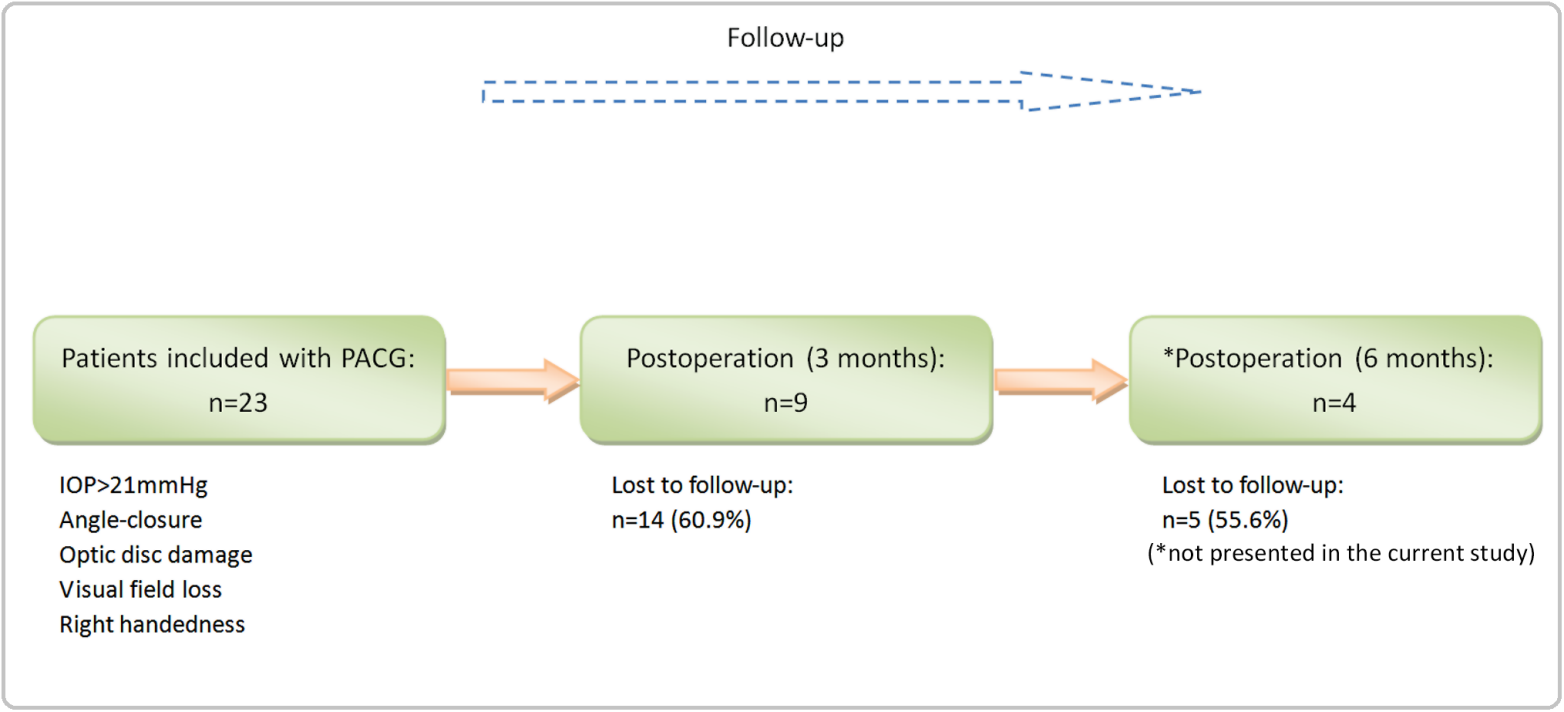
Schematic of recruiting procedures for the patients in this study. For the patients, we acquired brain MRI data and performed a complete ophthalmologic examination at three time points: before surgery and at three and six months after surgery. Due to poor compliance and a lack of transportation, we only included 9 patients three months after surgery and 4 patients six months after surgery.

This was a half-year-long longitudinal study, as illustrated in Fig 1. For each of the patients, we acquired brain MRI datasets and performed complete ophthalmologic examinations at three time points: before surgery and at three and six months after surgery. Before the surgery, patients received eyedrops of pilocarpine nitrate (MP Biomedicals, Solon, OH, USA), timolol maleate 0.2%/0.5% twice daily or brinzolamide (Alcon Laboratories, Inc.) to lower the IOP. After the surgery, they required no medicine to control the IOP.

The healthy controls were evaluated only at baseline (Fig 1). Due to poor compliance and a lack of transportation, only nine of these 23 PACG patients attended the examination and MRI scan at three months after surgery, and only four patients attended at six months after surgery. In this study, we attempted to compare the DC difference between the 23 pre-PACG patients and the controls and between the pre- and post-operative stages of the 9 post-PACG patients (two patients’ data were not conform to the standard of head motion, so at last the simple size was 7). Further details on brain activity changes of the 4 post-PACG patients at six months after surgery can be found in the supporting results (S1 Results). Table 2 lists the clinical variables of the post-PACG patients in the pre- and post-operative stages.

**Table 2:** Ophthalmological assessments of patients before (Pre-PACG) and after (Post-PACG) surgery.

PACG	Age (years old)	Gender	Duration	RNFL (μm)		Average C/D		Vertical C/D		IOP (mmHg)	
				Pre	Post	Pre	Post	Pre	Post	Pre	Post
sub1	50	M	2 d	95	92	0.49	0.44	0.48	0.39	38	15
sub2	40	F	1 y	80	96	0.74	0.66	0.69	0.66	30	16
sub3	55	F	2 d	97	95	0.69	0.74	0.59	0.69	55	16
sub4	63	F	7 d	95	96	0.67	0.62	0.62	0.63	54	18
sub5	44	F	2 d	107	96	0.6	0.64	0.55	0.58	33	12
sub6	45	F	4 d	61	53	0.94	0.91	0.93	0.91	53	21
sub7	67	F	8 y	61	71	0.78	0.77	0.85	0.87	35	17
sub8	69	M	1 m	62	60	0.83	0.83	0.82	0.79	47	14
sub9	52	M	3y	71	70	0.77	0.75	0.75	0.73	25	16
*p-*value				0.99		0.272		0.854		< 0.001**	

Abbreviation: RNFL, retina nerve fiber layer; A C/D, average cup to disc ratio; V C/D, vertical cup to disc ratio; IOP, Intraocular pressure; duration of disease (time from the first attack of the disease to being admitted to the study); M, male; F, female; d, day; y, year; m, month.

Note

* statistically significant differences.

### 2.2. MRI data acquisition

All MRI data were collected on a Siemens Trio 3.0 T scanner by implementing an 8-channel phased-array head coil in the First Affiliated Hospital of Nanchang University, China. Each subject lay in the supine position, with the head in a neutral position and fixed comfortably by a belt and foam pads during the MRI scanning. The resting-state functional MRI (R-fMRI) data were acquired using a gradient-echo echo-planer imaging (EPI) sequence with the following parameters: repetition time (TR) = 2,000 ms, echo time (TE) = 40 ms, flip angle = 90°, slice thickness/gap = 4.0/1 mm, field of view (FOV) = 240 mm × 240 mm, in-plane resolution = 64 × 64, 30 axial slices covering the whole brain, and 240 volumes acquired in 8 min. In addition, we acquired high-resolution brain structural images for each subject by using a T1-weighted 3D MP-RAGE sequence (TR = 1,900 ms, TE = 2.26 ms, flip angle = 9°, matrix = 256 × 256, FOV = 240 mm × 240 mm, thickness = 1.0 mm, and 176 sagittal slices).

### 2.3. fMRI data preprocessing

All preprocessing was performed using the Data Processing Assistant for Resting-State fMRI (DPARSF, http://www.restfmri.net), which is based on Statistical Parametric Mapping (SPM8) (http://www.fil.ion.ucl.ac.uk) and the Resting-state Data Analysis Toolkit (REST, http://www.restfmri.net). The first ten volumes of resting-state fMRI data were discarded for each subject to avoid any possible effects of scanner instability and adaptation of the subjects to the surroundings. The remaining 230 volumes acquired from each subject were corrected for differences in slice acquisition times. The resultant images were then realigned to correct for small movements between scans. Based on the recorded motion correction estimates, subjects with more than 2-mm maximum displacement in any of the x, y, or z directions or more than 2° of angular rotation in any axis for any of the 230 volumes were excluded from this study. Two post-PACG patients were excluded from the analyses based on this criterion. Individual T1-weighted structural images were co-registered to the mean of the realigned EPI images. The transformed structural images were then segmented into gray matter, white matter, and cerebrospinal fluid [30]. The Diffeomorphic Anatomical Registration Through Exponentiated Lie Algebra (DARTEL) tool [31] was used to compute the transformations from the individual native space to the MNI space and vice-versa. Resting-state MRI measures are sensitive to micro-head motions [32]; therefore, the Friston 24-Parameter Model [33] was used to regress head motion effects from the realigned data (the 24 parameters include 6 head motion parameters, 6 head motion parameters one time point before, and the 12 corresponding squared items), based on recent reports demonstrating that higher-order models benefit from the removal of head motion effects [32,34]. We further characterized the mean frame-wise displacement (FD), which considers measures of voxel-wise differences in motion in its derivation [35], as a measure of the micro-head motion of each subject [36]. To further reduce the effects of confounding factors, the signals from the white matter and cerebrospinal fluid, the mean time series of all voxels across the whole brain, and linear and quadratic trends were removed from the data via linear regression [36]. Temporal filtering (0.01–0.1 Hz) of the time series was then performed.

Using the DPARSF toolbox, we computed the voxel-specific head motion, including the values of voxel-specific frame-wise displacement (FDvox) and voxel-specific total displacement (TDvox) values for each subject. Group differences in the mean FDvox were calculated using a two-sample *t*-test, and the results were not significant. The mean FDvox was used as a covariate in the group comparisons of DC.

### 2.4. Degree centrality

The voxel-wise functional network was generated for each subject, for which we took each voxel as a node and inter-voxel correlations as the edge. Within the default brain mask provided by DPARSFA (in the MNI-152 standard space with 3×3×3 mm^3^ voxel size and resolution of 61×73×61), we used the preprocessed functional images to perform a voxel-wise correlation analysis. For each subject, we calculated *Pearson's* correlation between the time courses for any pair of voxels, resulting in a 70831-by-70831 correlation matrix. An undirected adjacency matrix was then obtained by setting a threshold to each correlation at *r* > 0.25 [23,26,37]. Based on the individual voxel-wise functional network, DC was calculated by counting the number of significant suprathresholded correlations (or the degree of the binarized adjacency matrix) for each subject. The voxel-wise DC map for each individual was converted into a *z*-score map using the following equation [23]:

$$Z_{i}=\frac{{DC}_{i}-mean\;(DC\;of\;all\;voxels\;in\;brain\;mask)}{std\;(DC\;of\;all\;voxels\;in\;brain\;mask)}$$ (1)

where *i* is the voxel index, *DC*_i_ is the DC value for the *i*-th voxel, std is the standard deviation, and *z*_i_ is the *z*-score for the *i*-th voxel. The *z*-score map was spatial smoothed with a Gaussian kernel of FWHM (full width at half maximum) of 6 mm.

### 2.5. Statistical analysis

The difference in age between the patients and controls was assessed with independent two-sample *t*-tests using SPSS (Statistical Package for the Social Sciences) 17.0 software (IBM, Armonk, NY).

The difference in *z*_i_ between the patients and controls was carried out with SPM8. We performed two-sample *t*-tests to examine the difference in the *z*-score between the pre-PACG patients and the controls and paired-sample *t*-tests to compare the difference in the *z*-score between the post- and pre-PACG patients.

All results were reported at the significant level of a threshold of two-tailed voxel-wise *p* < 0.01 and cluster level *p* < 0.05 with Gaussian Random Field (GRF) correction. Moreover, we performed a partial correlation analysis to assess the relationship between the mean DC values in all clusters showing significant differences and clinical variables in the patients. In the calculations, we regressed out the confounding covariates, including the mean FDvox, age, and gender.

## Results

### 3.1 Participant characteristics

The demographic measures in the pre-PACG patients were similar to (*p* > 0.05) those of the controls (Table 1). Compared with the pre-PACG group, the post-PACG patients showed a significant improvement in IOP (Table 2).

### 3.2. Degree centrality difference in PACG patients before and after surgery

**Fig 2:**
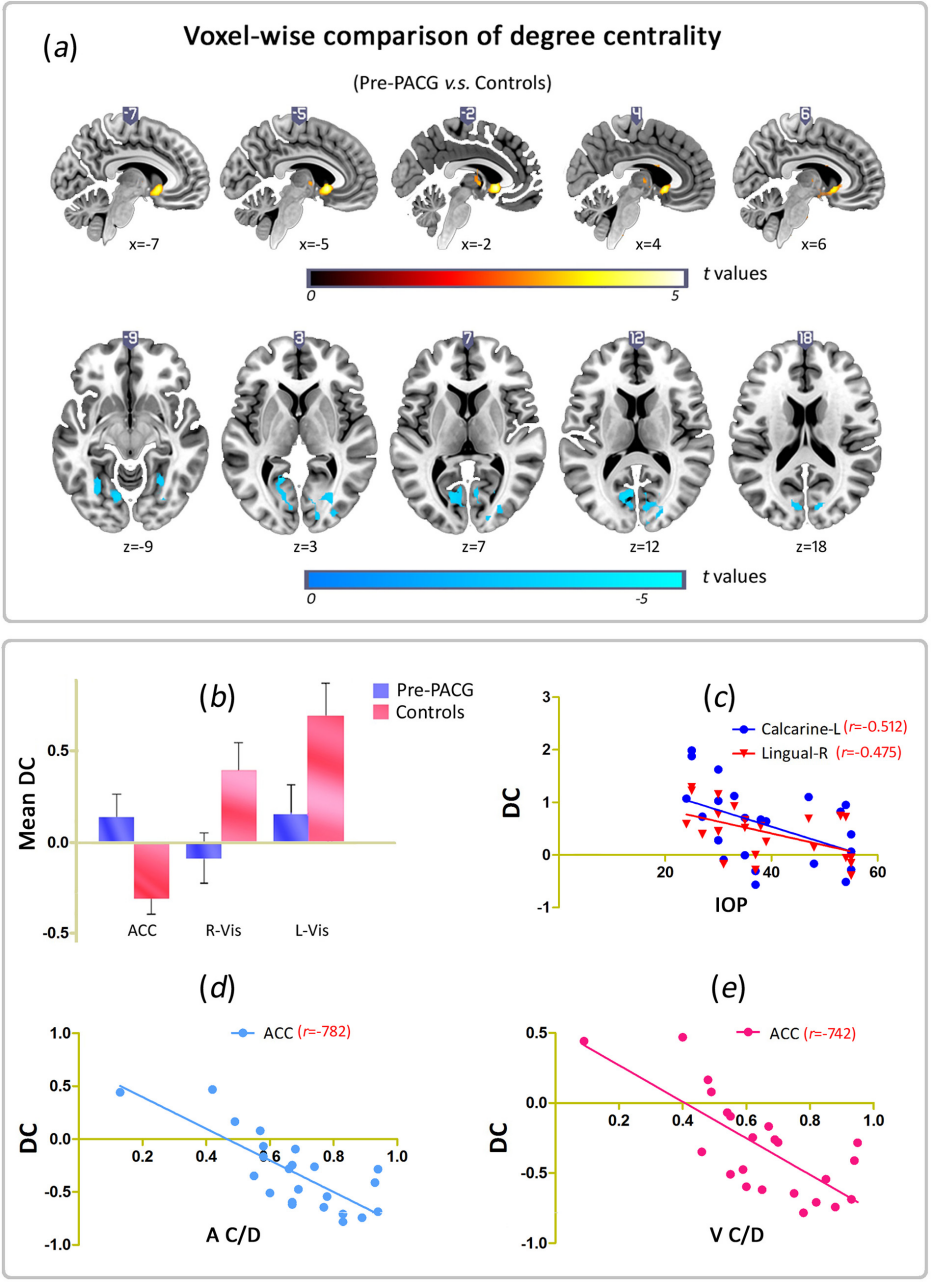
Voxel-wise comparison of DC between Pre-PACG and Controls & the relationships between changed DC areas and clinical variables. (*a*) Brain clusters showing a significant difference in degree centrality (DC) in the Pre-PACG patients compared with the Controls (cluster-level *p* < 0.05, GRF correction). Hot color (cool) indicates significant increased (decreased) DC in the Pre-PACG patients. (*b*) Bar plot of DC for the significant clusters in Pre-PACG *v*.*s*. Controls. DC, degree centrality; ACC, anterior cingulate cortex; L(R), left (right) hemisphere; Vis, visual. (*c*) DC values in the right lingual gyrus (R Ling, *r* = -0.512, *p* < 0.05) and left calcarine (L Cal, *r* = -0.475, *p* < 0.05) are significantly negatively correlated with the intraocular pressure (IOP) in the Pre-PACG patients. *(d*) DC values in the left anterior cingulate cortex (ACC) were significantly negatively correlated with A C/D (*r* = -0.782, *p* < 0.05) in the Pre-PACG patients. (*e*) DC values in the left anterior cingulate cortex (ACC) were significantly negatively correlated with V C/D (*r* = -0.741, *p* < 0.05) in the Pre-PACG patients.

**Fig 3:**
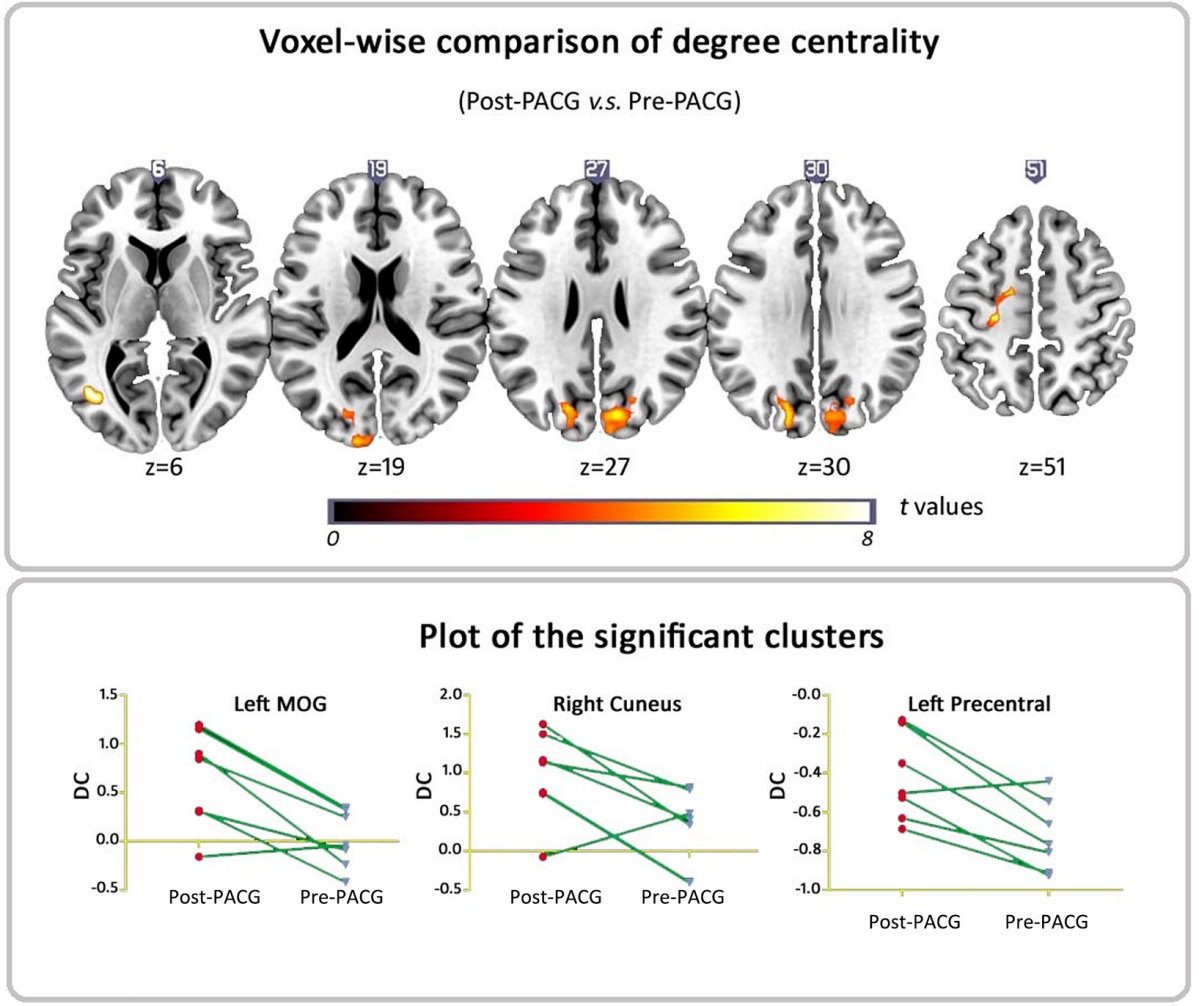
Voxel-wise comparison of DC between Post- and Pre-PACG patients. Areas of significant (*p* < 0.05, GRF corrected) DC difference between Post- and Pre-PACG patients. Hot (cool) color represents higher (lower) DC in the Post-PACG group compared with the Pre-PACG group. a) Axial view and b) DC values of significant different clusters (left MOG and right cuneus and left precentral gyrus) between Post- and Pre-PACG patients. Abbreviation: MOG, middle occipital gyrus.

Fig 2-A and S1 Fig show brain clusters with a significant difference in DC in the pre-PACG patients compared with the controls (voxel *p* < 0.01 and cluster-level *p* < 0.05, GRF correction) (Fig 2-A and S1 Fig). Fig 3 and S1 Fig show clusters with a significant difference in DC between the post- and pre-PACG groups (voxel *p* < 0.01 and cluster-level *p* < 0.05 with GRF correction) (Fig 3 and S1 Fig).

Compared with the controls, the pre-PACG patients showed a significantly decreased DC in two clusters (Fig 2-A and 2-B). One cluster was located in the left lingual gyrus and extended to the calcarine, cuneus, and fusiform. The other cluster was located in the right middle occipital gyrus and extended to the cuneus and lingual gyrus (Table 3).

**Table 3:** Brain areas with significantly different DC between Pre-PACG patients *v*.*s*. Controls and between Post- *v*.*s*. Pre-PACG patients (*p* < 0.05, GRF corrected).

	Brain regions	MNI coordinates			Location	Cluster size (No. of voxels)	*t-*value
		x	y	z			
Pre-PACG	left lingual gyrus	-15	-54	-3	BA 18, 19, 30	305	-5.61
*v*.*s*.	right middle occipital gyrus	30	-72	0	BA 18, 19	221	-4.16
Controls	left anterior cingulated cortex	-3	15	-3	BA 24, 25	227	5.01
Post-PACG	right cuneus	18	-72	36	BA 18, 19, 7	363	8.89
*v*.*s*.	left middle occipital gyrus	-45	-66	3	BA 19, 37	73	10.38
Pre-PACG	left precentral gyrus	-24	-21	51	BA 6, 3	67	7.95

BA, Brodmann's area; (x, y, z), coordinate of peak location in the MNI space; *t*, statistical value of peak voxel; *p* < 0.05, Gaussian Random Field (GRF) theory correction; L (R), left (right) hemisphere.

We further examined the change in DC between post- and pre-PACG patients. Fig 3 and S1 Fig show significantly increased DC in three clusters, the bilateral visual cortices and left precentral gyrus in the post-PACG patients. The cluster located in the right visual cortex was approximately in the same region found at the evaluation of pre-PACG patients compared with the controls. That is, after surgery, the DC of the right visual cortex significantly increased. In addition, we found significantly increased DC in the left precentral gyrus in the post-PACG group compared with the pre-PACG patients. This cluster peaked in the left precentral gyrus and extended to the left middle frontal cortex and the left postcentral gyrus. Detailed information for these clusters is listed in Table 3.

To test that our findings were robust or not dependent upon the selection of threshold for network construction, we repeated the network analysis using a range of correlation thresholds (*r* = 0.15, 0.20, 0.30 and 0.35). And we found that the selection of the threshold would not influence the results significantly. The results are showed in the supplement materials (S2 Fig).

### 3.3. Correlation between clinical variables and DC

For the six clusters listed in Table 3, we analyzed the correlations between the DC value and clinical variables in the patients. In the calculations, we controlled age and gender as covariates. Fig 2 shows the significantly negative correlation between the DC values in the bilateral visual cortices and the IOP score in the pre-PACG patients. Specifically, the DC values in the right lingual gyrus (R Ling, *r* = -0.512, *p* < 0.05) and the left calcarine (L Cal, *r* = -0.475, *p* < 0.05) were significantly negatively correlated with the IOP in the pre-PACG patients (Fig 2-C). In the left anterior cingulate cortex (ACC), the pre-PACG patients showed that the DC value was significantly negatively correlated not only with A C/D (*r* = -0.782, *p* < 0.05) (Fig 2-D) but also with V C/D (*r* = -0.741, *p* < 0.05) (Fig 2-E). However, no significant relationship was found between the duration of disease and the DC value in any of the changed clusters.

## Discussion

We investigated the functional network centrality changes measured by resting-state fMRI in PACG patients before and after surgery. Consistent with our hypothesis, we observed network centrality changes involved in visual and cognition-emotional processing regions. Specifically, our results showed significantly decreased DC in bilateral visual cortices but increased DC in the ACC and caudate in PACG patients.

The pathology of glaucoma is featured in the death of RGCs [3]. The death of RGCs can lead to a loss of neural cells in the LGN, which is the major vision center that relays information from the eye to the visual cortex, and the visual cortex is secondarily affected. Previous evidence has indicated that Wallerian and transsynaptic degeneration play a causative role in POAG [38]. However, in PACG, smaller anterior segment dimensions serves as the hallmark [39]. Prerequisite anatomical risk factors include shallower anterior chamber depth (ACD), the cardinal feature associated with increased susceptibility to PACG, an anterior lens position and short axial length [5,7]. Genetic and environmental influences may jointly impact the disease’s emergence and development [2,6,8]. Degree centrality can be considered the ability for information integration, and high DC may serve as a hub for the traffic operation of functional networks, superior information propagation and may thus contribute to efficient information flow [23,37]. In this framework, the decreased DC of the bilateral visual cortices observed in our study may be an expression of decreased visual sensory information input and degenerative “hubness” associated with PACG. Vision loss or eye conditions related to changes in cortical thickness [12,40,41], density, and volume [11,42,43] have been observed in humans associated with both POAG and blindness. A considerable body of literature has attempted to understand the mechanisms of sensory integration and the interplay that underlies neuroplasticity after visual deprivation [44]. These literatures includes decreased topology and visual centrality in early blindness [45,46], with lowered but near the normal degree connectivity of visual regions in the adolescent-blind, and late-blind subgroups [47]. Contrary to age-related macular degeneration, which primarily affects the central visual field, glaucoma is reported as being implicated in the peripheral visual field [48]. In experimental glaucoma, degenerative changes are observed in the optic nerve, pathways in the LGN, and the visual cortex. For example, Chan et al. [49] employed proton magnetic resonance spectroscopy (MRS) and found an altered metabolism of Cho-containing compounds in the visual cortex, which suggested potentially transsynaptic degeneration and visual cortical dysfunction. Our results of decreased DC in the bilateral visual cortices agree with the notion that glaucoma imposes anterograde neurodegenerative changes in the visual cortex and that this decreased DC may reflect anterograde neuronal degeneration of the visual cortex.

In addition to decreased DC in the visual cortex, we found significant increased DC in the ACC and caudate (Fig 3). ACC is believed to be the pinnacle of brain evolution in humans: flexible but vulnerable, primarily engaged in cognition/emotional control and involved in several psychiatric disorders [50,51]. Several lines have been directed at the role of ACC in glaucoma, although these lines remain unclear. Increased local coherence of low frequency resting BOLD time courses [22], lower grey matter volume [14], and higher functional connectivity in the medial part of the executive network [14] in the ACC in POAG were found. However, carefully controlling for patients, we only found a negative association between the ACC degree centrality and A C/D, V C/D in the current study. Because of a lack of mood ratings, this finding suggests the necessity for assessing the mood scale for use as covariates when investigating brain alterations in glaucomatous populations, as previous research has suggested a higher prevalence of anxiety and depression in primary glaucoma patients, especially in PACG patients in China [52,53]. The caudate, along with the putamen and globus pallidus, makes up the basal ganglia and is implicated in a range of functions, including the regulation of cortical excitability and sensory processing [54]. Glaucoma patients are frequently reported to have impaired proprioception under somatosensory perturbations [55,56,57]. Two main factors may exist: one is impairment of the peripheral visual system, and the other is the preoperative drug administration (predominantly beta-blockers); they both play a vital role in maintaining posture and balance. The caudate recruitment of DC observed in the current study may be associated with altered proprioception and somatosensory processing. Speculatively, alterations in DC in the ACC and caudate may echo a brain response to glaucomatous insult and resilience to stress and depressive loads.

Interestingly, we tentatively examined the postoperative transformation of DC to test the neuroplastic trajectories of surgery over 3 months and found heightened DC in the visual cortex and primary sensory and supplementary motor areas. This postoperative enhancement, accompanied by postoperatively lower IOP and ease of symptoms, may indicate that the postoperative plasticity of functional network centrality in the visual cortex occurs and that this neuroplasticity underlies improved behaviors. Whereas the glaucomatous changes in the visual pathway are generally considered transsynaptic/anterograde degeneration [2,13], one possible explanation for this result may be the restoration process. Carefully screening the patients, we found six of the follow-up post-PACG patients had a sudden onset of total angle closure; thus, there was not enough time to change profoundly.

## Limitations

There are several limiting factors that should be acknowledged. First, there was a lack of mood items. PACG patients seem to have higher depression and anxiety scores compared with controls. Although none of our participants had an affective or anxiety disorder, a subtle impact of subclinical depression or anxiety on DC cannot be excluded. Second, this was primarily a cross-sectional study, and follow up was limited. Therefore, conclusions on the neuroplastic trajectories of surgery can not be well drawn, and larger longitudinal samples are needed to refine our understanding of the potential normalized processes. Third, preoperative drug administration (i.e., timolol maleate, brinzolamide, mannitol) is used to lower IOP, but its impacts on the brain intrinsic dynamics are unclear. Lastly, the duration of all PACG patients varied largely, and the brain activity alterations may not be the same within different disease stages.

## Conclusions

In summary, our study suggests that PACG may contribute to decreased functional centrality in visual and cognition-emotional processing regions and that alterations in visual areas do not seem parallel to the time of onset. The operative outcome of functional centrality may depend on different subgroups of glaucoma. The framework facilitates further studies and understanding of the pathophysiology of PACG.

## Supporting Information

S1 Fig Voxel-wise comparison of DC between Pre-PACG *v*.*s*. Controls and Post- *v*.*s*. Pre-PACG patients visualized with the BrainNet Viewer. Row (a) and (b) shows the different brain areas of DC between Pre-PACG *v*.*s*. Controls and Post- *v*.*s*. Pre-PACG, respectively. Cool color (blue) indicates the decreased DC areas and the hot color (red) indicates the opposite. Left in the figure indicates the left side of the brain.
(TIF)

S2 Fig Voxel-wise comparison of DC between Pre-PACG *v*.*s*. Controls and Post- *v*.*s*. Pre-PACG patients with different thresholds. The four boxes demonstrate the results with different thresholds (*r* = 0.15, 0.20, 0.30 and 0.35) respectively. Row (a) and (b) in each box shows the different brain areas of DC between Pre-PACG *v*.*s*. Controls and Post- *v*.*s*. Pre-PACG, respectively. Cool color (blue) indicates the decreased DC areas and the hot color (red) indicates the opposite. Left in the figure indicates the left side of the brain. And the results in the paper are not influenced with different thresholds.
(TIF)

S1 Results Appendix to the manuscript. (DOC)
