# Supplementary figures and images for: Network Centrality of Resting-State fMRI in Primary Angle-Closure Glaucoma Before and After Surgery
Source: PLoS One. 2015 Oct 27;10(10):e0141389. doi: 10.1371/journal.pone.0141389 (PMC4624709; doi:10.1371/journal.pone.0141389)

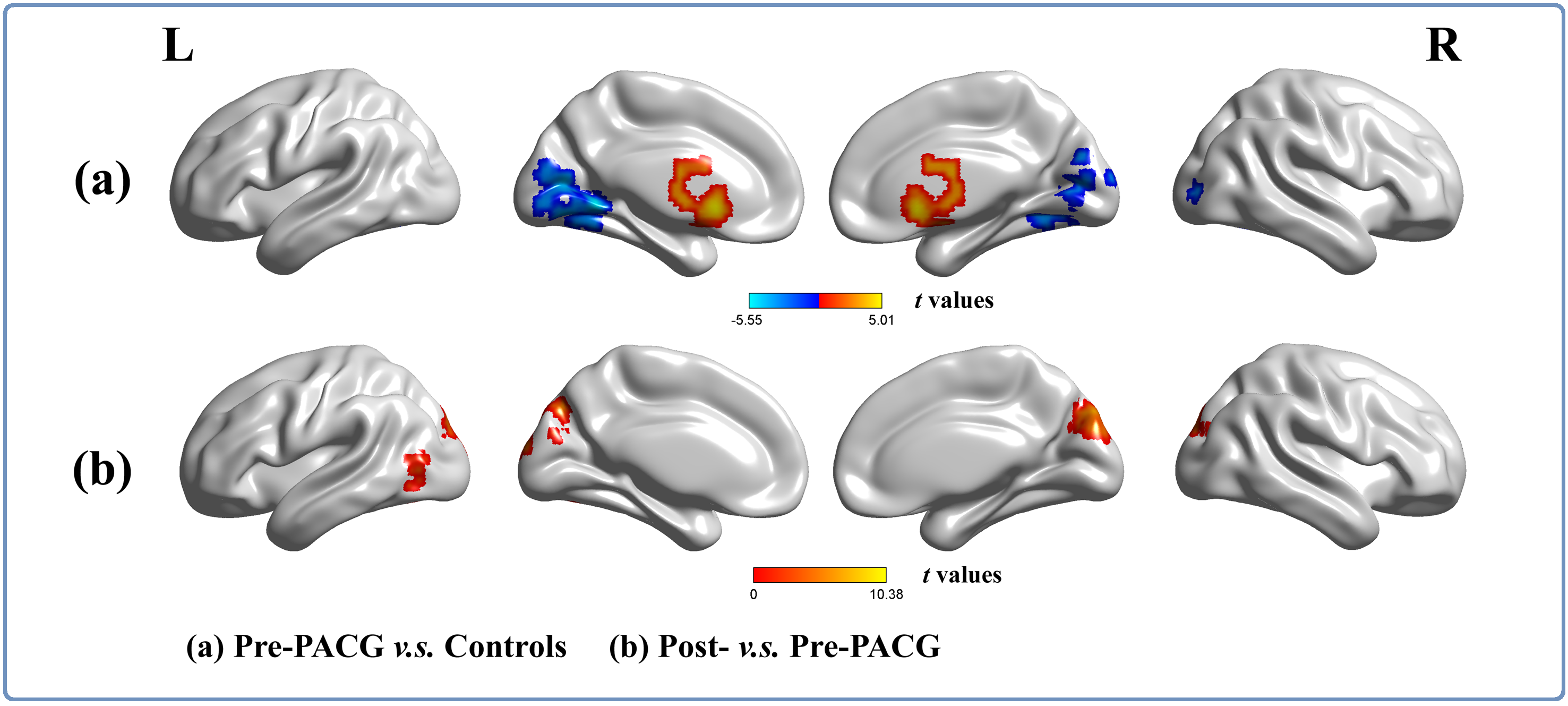

Supplement: S1 Fig — Row (a) and (b) shows the different brain areas of DC between Pre-PACG v.s. Controls and Post- v.s. Pre-PACG, respectively. Cool color (blue) indicates the decreased DC areas and the hot color (red) indicates the opposite. Left in the figure indicates the left side of the brain. (TIF) [file pone.0141389.s001.tif]

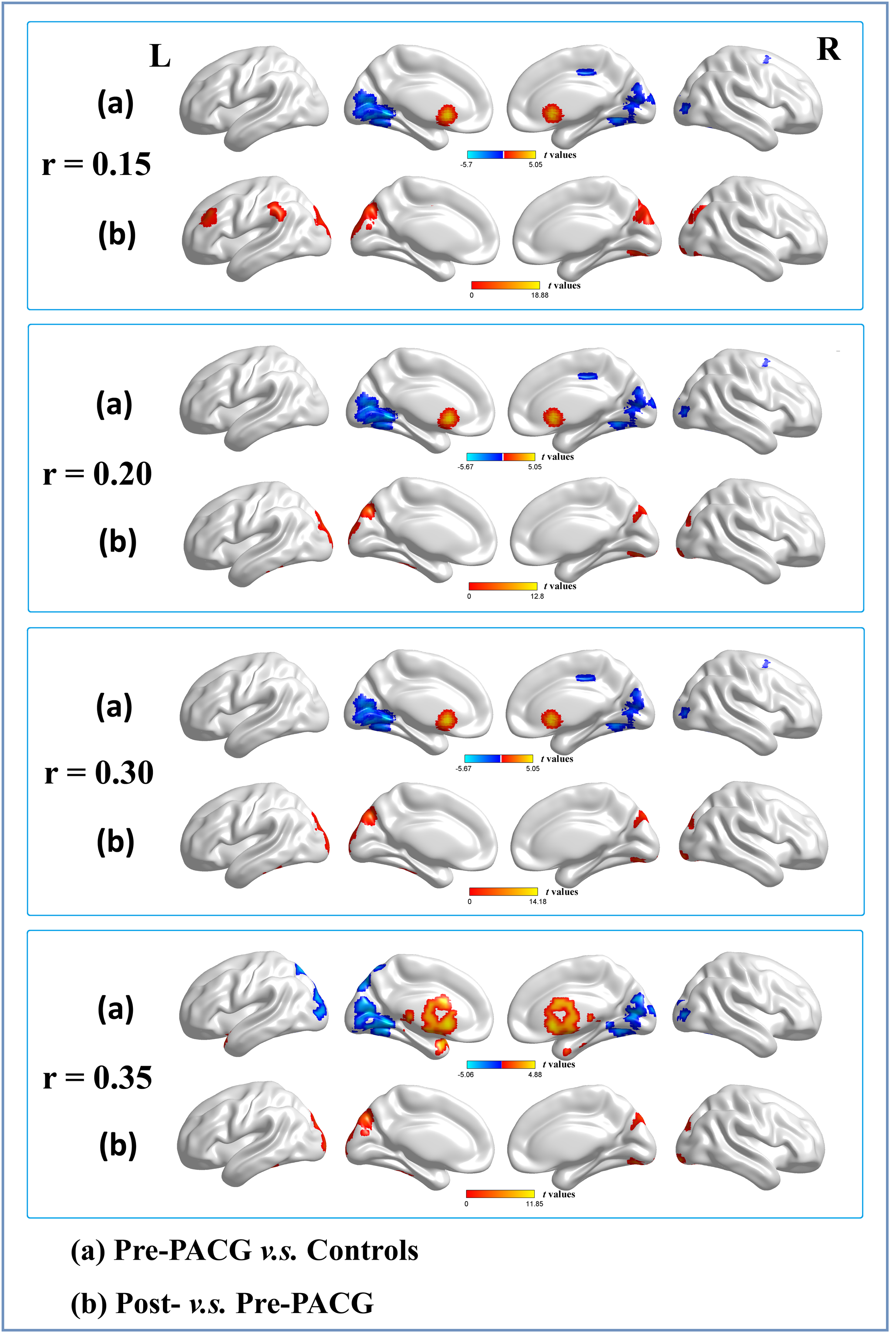

Supplement: S2 Fig — The four boxes demonstrate the results with different thresholds (r = 0.15, 0.20, 0.30 and 0.35) respectively. Row (a) and (b) in each box shows the different brain areas of DC between Pre-PACG v.s. Controls and Post- v.s. Pre-PACG, respectively. Cool color (blue) indicates the decreased DC areas and the hot color (red) indicates the opposite. Left in the figure indicates the left side of the brain. And the results in the paper are not influenced with different thresholds. (TIF) [file pone.0141389.s002.tif]
