# Supplementary material for: Network Centrality of Resting-State fMRI in Primary Angle-Closure Glaucoma Before and After Surgery
Source: PLoS One. 2015 Oct 27;10(10):e0141389. doi: 10.1371/journal.pone.0141389 (PMC4624709; doi:10.1371/journal.pone.0141389)
Supplement: S1 Results — (DOC) [file pone.0141389.s003.doc]

**S1 Results. The brain activity changes of the patients 6 months after surgery compared with their previous data of 3 months after surgery as well as matched normal controls. (During the revising period, we collected one more patient 6 months after surgery, so the total simple size is 5.)**

The patients re-scanned 6 months after successful surgery showed no evolutive changes in visual cortex compared with their previous data of 3 months after surgery, but slightly increased DC (marginal statistics) in the ventral medial prefrontal cortex(BA10) and left inferior parietal lobule(BA40). Besides, compared with the matched controls, the patients demonstrated increased DC in the ventral medial prefrontal cortex, orbitofrontal cortex and precuneus, but no statistically significant difference along the visual areas were found. As we know, the inferior parietal lobule takes parts in multidimensional sensory information processing, and the orbitofrontal cortex involves in self-evaluation, rewarding and decision-making. So we speculate that: (1) The patients 6 months after surgery show no difference of DC in visual cortex compare to their previous state of 3 months after surgery, that may due to the short time which is not enough for the evolvement of visual areas. (2) The patients 6 months after surgery show no difference of DC in visual cortex compare to controls, that may reflect the neuroplasticity by surgery. (3) The patients exist functional alterations in some extra-visual areas, which may suggest the visual-perception interaction changes. Since the small simple size, the trend of trajectory after surgery may provide a preliminary clue to exploring the potential mechanism of glaucoma.
